# G9a-mediated repression of CDH10 in hypoxia enhances breast tumour cell motility and associates with poor survival outcome

**DOI:** 10.7150/thno.41453

**Published:** 2020-03-15

**Authors:** Francesco Casciello, Fares Al-Ejeh, Mariska Miranda, Greg Kelly, Eva Baxter, Karolina Windloch, Frank Gannon, Jason S Lee

**Affiliations:** 1 QIMR Berghofer Medical Research Institute, Herston Rd, Herston, QLD 4006, Australia.; 2 School of Biomedical Sciences, Queensland University of Technology, Kelvin Grove, QLD 4059, Australia.; 3 School of Medicine, University of Queensland, Herston, QLD 4006, Australia.; 4 Currently at Cancer Research Center, Qatar Biomedical Research Institute, Doha, Qatar.

**Keywords:** G9a, hypoxia, CDH10, metastasis, breast cancer

## Abstract

**Rationale**: Epigenetic mechanisms are fundamental processes that can modulate gene expression, allowing cellular adaptation to environmental conditions. Hypoxia is an important factor known to initiate the metastatic cascade in cancer, activating cell motility and invasion by silencing cell adhesion genes. G9a is a histone methyltransferase previously shown to accumulate in hypoxic conditions. While its oncogenic activity has been previously reported, not much is known about the role G9a plays in the hypoxia-mediated metastatic cascade.

**Methods**: The role of G9a in cell motility in hypoxic condition was determined by inhibiting G9a either by short-hairpin mediated knock down or pharmacologically using a small molecule inhibitor. Through gene expression profiling, we identified CDH10 to be an important G9a target that regulates breast cancer cell motility. Lung metastasis assay in mice was used to determine the physiological significance of G9a.

**Results**: We demonstrate that, while hypoxia enhances breast cancer migratory capacity, blocking G9a severely reduces cellular motility under both normoxic and hypoxic conditions and prevents the hypoxia-mediated induction of cellular movement. Moreover, inhibition of G9a histone methyltransferase activity in mice using a specific small molecule inhibitor significantly reduced growth and colonisation of breast cancer cells in the lung. We identify the type-II cadherin *CDH10* as being a novel hypoxia-dependent gene, directly repressed by G9a through histone methylation. CDH10 overexpression significantly reduces cellular movements in breast cancer cell lines and prevents the hypoxia-mediated increase in cell motility. In addition, we show that *CDH10* expression is prognostic in breast cancer and that it is inversely correlated to *EHMT2* (G9a) transcript levels in many tumor-types, including breast cancer.

**Conclusion**: We propose that G9a promotes cellular motility during hypoxic stress through the silencing of the cell adhesion molecule CDH10 and we describe *CDH10* as a novel prognostic biomarker for breast cancer.

## Introduction

Hypoxia is defined as a state of reduced oxygen tension in a tissue. Hypoxic tumour microenvironment often occurs in solid tumors and clinical studies have shown that that it directly associates with a more aggressive phenotype by increasing the risk of metastasis, hence correlating with poor prognosis 1-4. Importantly, metastasis is known to be the primary cause of treatment failure in patients with solid tumors 5,6. Previous studies have described the response to hypoxia to be mainly dependent on the hypoxia inducible factor (HIF) family of transcription factors 7,8. More recently, we and others have demonstrated that epigenetic enzymes also play an important role in regulating gene expression under this condition 9,10.

G9a, encoded by the *EHMT2* gene is a histone methyltransferase which catalyses mono and di-methylation of histone 3 lysine 9 (H3K9), a modification associated with gene repression 10,11. In cancer, G9a is upregulated in a variety of neoplasms, including lung, colon, ovarian, oesophageal squamous cell and hepatocellular carcinomas, and correlates with tumor aggressiveness and poor patient prognosis 10,12-16. We have previously shown that G9a protein stability is increased in hypoxia in a similar manner to that of HIF-α proteins, leading to hypermethylation of H3K9 10. In addition, we have also previously demonstrated that G9a can directly methylate other non-histone proteins under hypoxia. In this context, methylation of the chromatin- remodelling factors Reptin and Pontin modulates a subset of hypoxia-responsive genes and thereby influences the ability of cells to respond to oxygen deprivation 17,18.

The association between metastasis and tumor hypoxia is believed to be mediated by changes in the expression of cell adhesion molecules, such as EpCAM (epithelial cell adhesion molecule) and cadherins, which are critical for cell adhesion. The loss of these proteins in hypoxia allows tumor cells to detach from the primary site, migrate and establish metastases in distant areas of the body 19-21. This process is a hallmark of the epithelial to mesenchymal transition (EMT). During EMT, it is believed that cells undergo a switch between epithelial and mesenchymal pro-migratory cadherins, thus leading to enhanced tumor aggressiveness 22. Therefore, EMT is recognised to play a major role in the promotion of metastasis and recent evidence demonstrated that hypoxia can actively stimulate EMT in cancer 23,24.

Loss of cadherin expression has been demonstrated to be important in EMT induction and cancer metastasis. Cadherins are a class of calcium- dependent transmembrane proteins, subdivided into different groups: the classical, desmosomal, protocadherins and unconventional (or ungrouped) cadherins. In addition, cadherins are also distinguished between type I and type II, where type II lack the histidine- alanine-valine (HAV) cell adhesion recognition sequence which distinguishes type I 25,26. Hypoxic stress is known to mediate the silencing of E-cadherin in various cancer types via HIFα-dependent upregulation of the transcription factor SNAIL 27,28. Loss of E-cadherin correlates with tumor invasiveness indicating that the expression of specific cadherins influences the aggressive phenotype in cancer 27. CDH10 is a type II cadherin thought to be predominantly expressed in the brain. However, mutations and loss of expression of this protein have been observed in multiple cancer types, including gastric, colorectal, pancreatic, endometrial and lung 29-31. Recently, CDH10 has also been suggested to act as a tumor suppressor in lung cancer by inhibiting cell motility *in vitro* 32. However, the link between CDH10 and metastasis has not been extensively studied to date, with only one report of CDH10 mutation being frequent in metastatic endometrial cancer 33.

In the present study, we demonstrate that G9a accumulation in hypoxia leads to the transcriptional repression of *CDH10*. We show that *CDH10* expression is directly regulated by G9a under reduced oxygen pressure and that its loss enhances breast cancer cell motility, even in metastatic cell lines that have already lost expression of E-cadherin and EpCAM. In addition, we demonstrate that *CDH10* expression is associated with improved relapse‑free survival in breast cancer, indicating that CDH10 acts as a metastasis suppressor and its downregulation is an integral part of hypoxia-mediated EMT in breast cancer.

## Results

### Inhibiting G9a reduces cell motility in breast cancer cell lines and attenuates the hypoxia-mediated response

**Figure 1:**
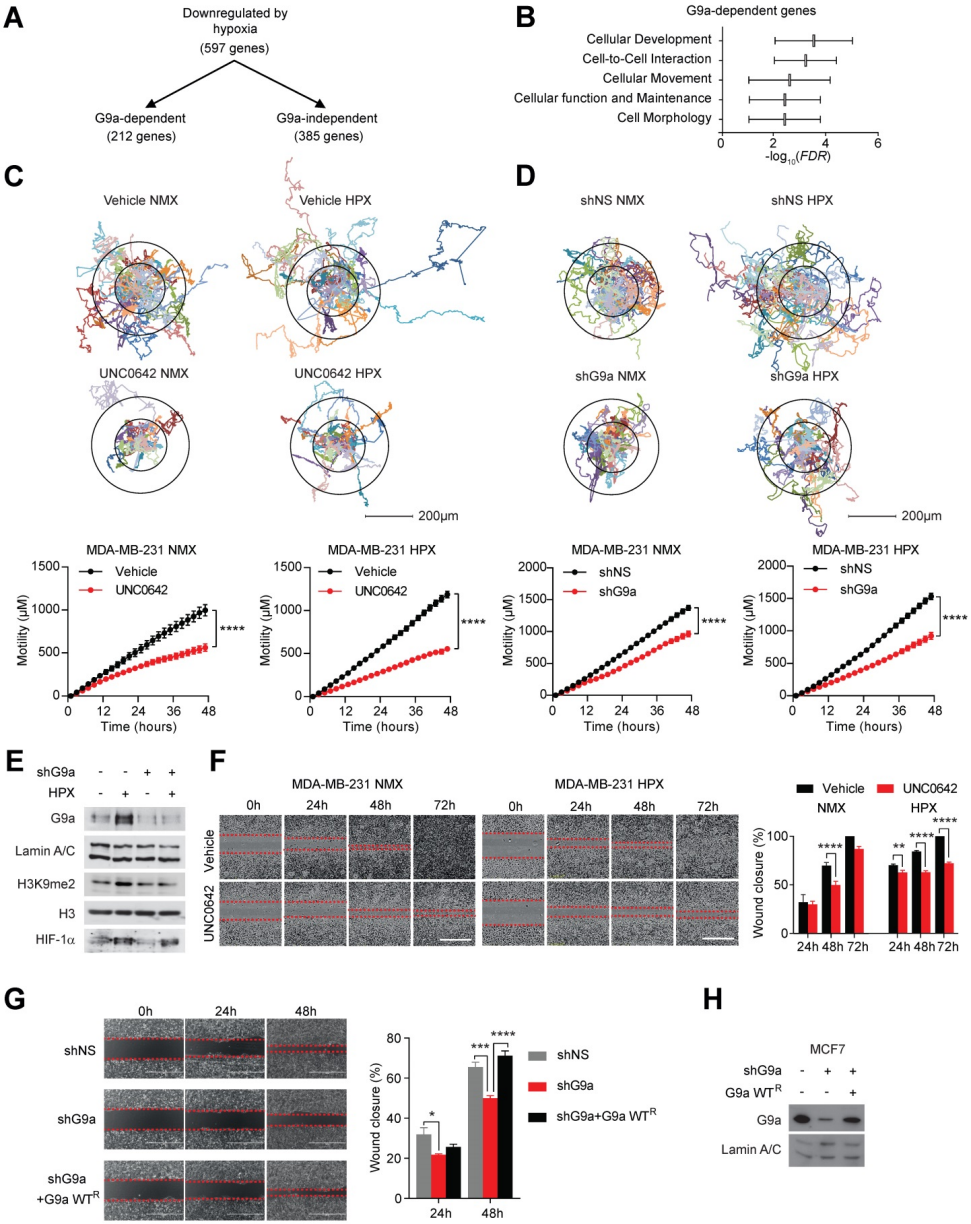
** G9a inhibition reduces cell motility in breast cancer cell lines.** G9a inhibition represses breast cancer cell motility *in vitro*. **(A)** Microarray analysis of differentially expressed genes comparing hypoxia-responsive genes from MCF7 cells expressing shNS and shG9a identifies a subset of 212 genes downregulated in hypoxia in a G9a-dependent manner. **(B)** Ingenuity Pathway Analysis results of the 212 G9a-dependent genes. **(C)** Evaluation of the migratory distance covered by MDA-MB-231 treated with UNC0642 (5 μM) or **(D)** transfected with either vector or shG9a. Results were evaluated through real-time imaging using the HoloMonitor M4, taking pictures every 10 minutes for 48 hours, in three independent experiments. An average of 20 cells per condition is shown for the representative circular displacement images. **(E)** Western blot analysis of G9a, H3K9me1 and H3K9me2 in MDA‑MB-231 transfected with vector or shG9a and incubated in hypoxia for 24 hours. HIF1α was included as positive control for hypoxic conditions. H3 and Lamin A/C were used as loading control **(F)** Scratch wound assay for MDA-MB-231 breast cancer cells treated with 5 μM UNC0642, under both normoxic (21% O_2_) and hypoxic (1% O_2_) conditions. Results were evaluated by real-time imaging performed by the IncuCyte Zoom every 24 hours and wound closure was quantified using ImageJ. Scale bar represents 500 μm. **(G)** Scratch wound assay of MCF7 breast cancer cells following G9a KD and G9a reconstitution. Scale bar represents 500 μm. **(H)** Western blot analysis of MCF7 cells transfected with shG9a and reconstituted with WT G9a. Data are represented as mean ± SEM of three independent experiments (unpaired, non-parametric Student's t-test, **p<0.005, ****p<0.0001).

We have previously provided a detailed molecular mechanism for G9a protein stabilization in hypoxia and identified a subset of hypoxia-responsive genes directly repressed by G9a in breast cancer cells 10. Amongst 597 genes repressed in hypoxia, 212 were found to be G9a-dependent (Figure 1A). We performed pathway analysis using Ingenuity Pathway Analysis (IPA) and found that a significant number of these G9a-dependent repressed genes were associated with processes influencing cell movement and cell motility (Figure 1B). We therefore examined whether genetic or pharmacologic inhibition of G9a impacts breast cancer cell motility in a poorly metastatic estrogen receptor positive (ER+) cell line (MCF7), and a highly metastatic, estrogen receptor negative (ER-) (MDA-MB-231) cell line. Importantly, MDA-MB-231 has been previously described to have lost the expression of the cell-adhesion molecules E-cadherin and EpCAM 34-36. Cells were treated with a small molecule inhibitor of G9a, UNC0642 (2 and 5 μM), and motility was analysed by performing holographic time-lapse imaging for up to 96 hours (HoloMonitor M4, Phase). Holographic microscopy allowed precise single cell-tracking and quantification of the total distance travelled. Results demonstrated that UNC0642 treatment was able to significantly reduce cellular motility, with the highest effects observed at 5 μM in both cell lines (Figure S1A). At this concentration, effects on cellular movements were clear as early as at 48 hour time point. The effects of inhibiting G9a on cell motility were then investigated under hypoxic (1% O_2_) conditions in the two cell lines. Analysis revealed that the hypoxic environment was able to enhance cell motility in both cell lines, increasing the average distance travelled (Figure S1B and C). In contrast, G9a inhibition in MDA-MB-231 and MCF7 cells led to a significant reduction in cell motility compared to the vehicle treated cells in both normoxic and hypoxic conditions (Figures 1C and S2A). To confirm that the observed phenotype was directly associated with the modulation of G9a activity, a similar experiment was performed following G9a knock down (KD). Cells were transfected with short hairpin constructs targeting G9a (shG9a) or a non-silencing control (shNS), resulting in a reduction in cellular motility comparable to that observed with inhibitor treatment (Figures 1D and E, S2B and C). To further confirm the role of G9a in cellular motility, a scratch wound assay was performed in the presence of UNC0642 under normoxic and hypoxic conditions in MDA-MB-231 and MCF7. The scratch wound healing assay recapitulated the effects previously observed through single cell tracking, where G9a inhibition reduced cell movements in the two breast cancer cell lines (Figures 1F and S2D). The inhibitory effect of UNC0642 on cellular migration was significantly greater in hypoxic conditions, where cells were not able to close the wound even after 72 hours. To determine the functional role of G9a protein, additional scratch wound assay was performed in MCF7 following G9a KD and reconstitution with G9a (Figures 1G and H). G9a KD inhibited wound closure while reconstitution with wild type (WT) G9a was able to restore cellular motility, further supporting the role of G9a in regulating breast cancer motility phenotype. In addition, the effect of inhibiting G9a on the invasive potential of breast cancer cells has also been investigated through matrigel invasion assay (8 mg/ml of matrigel) (Figure S2E). While poorly invasive MCF7 cells could not close the wound, treatment with UNC0642 significantly suppressed invasion of MDA-MB-231 cells, demonstrating that G9a activity is also important for the invasive potential of breast cancer cell lines. Because pharmacologic inhibition of the enzyme, as well as its knock down, have been previously reported to induce cell death in cancer cell lines, cell death was analysed using a specific fluorescent cell-impermeable stain (NucGreen) that binds DNA in dead cells (Molecular Probes, Life Technologies). As shown in Figure S3, no increase in fluorescence was observed with either G9a inhibitor treatment (5 μM) or G9a KD, suggesting that the inhibitory effects observed on cellular motility are not influenced by the induction of cell death. Together, these results demonstrate that hypoxia enhances cell motility and that G9a inhibition is sufficient to block this phenotype under both normoxic and hypoxic conditions in breast cancer cell lines.

### G9a inhibition reduces lung colonization and metastatic burden in a mouse model of breast cancer

**Figure 2:**
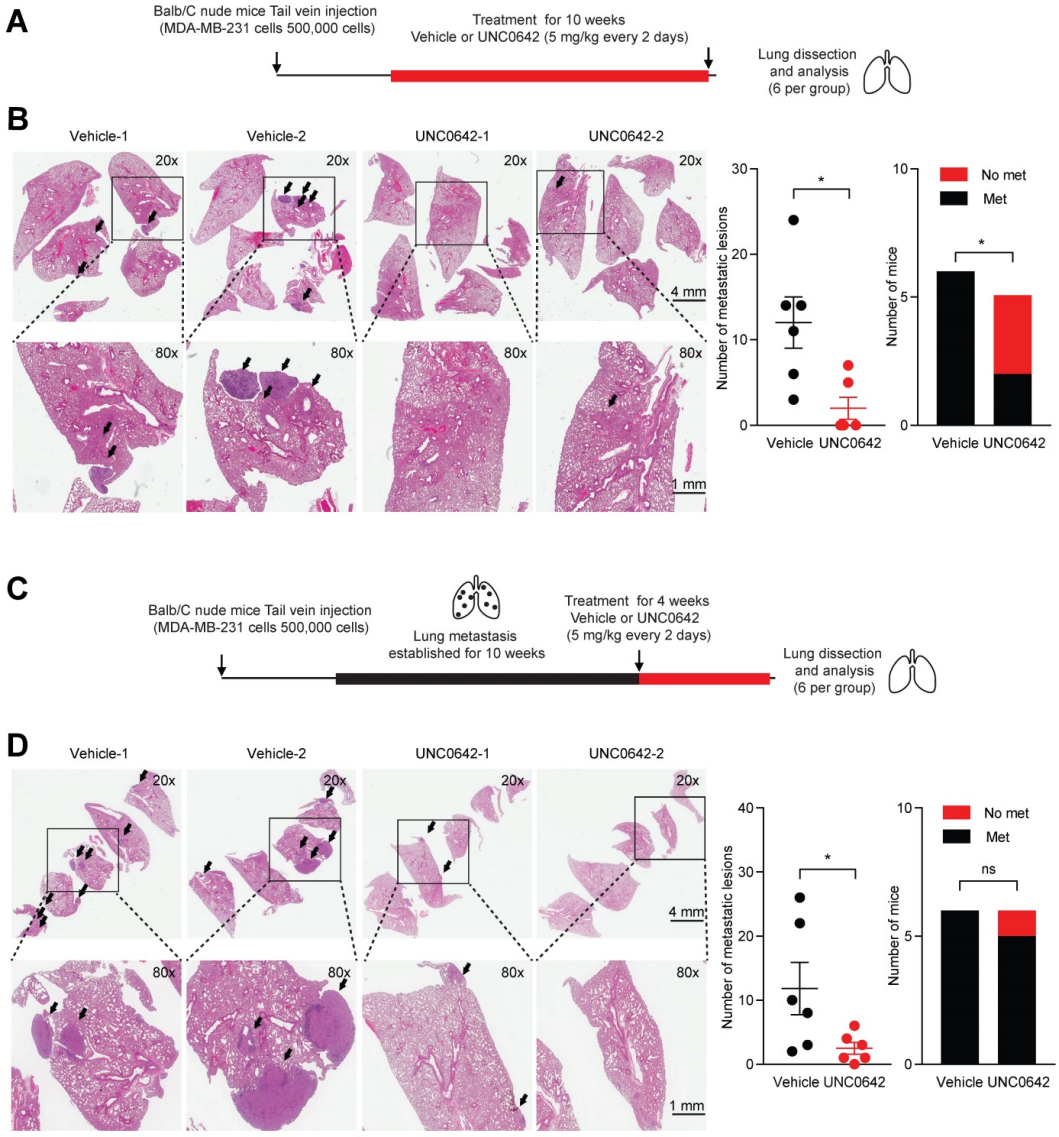
** G9a inhibitor reduces lung metastasis colonization and growth.** UNC0642 treatment impacts lung metastasis formation and inhibits growth of the metastatic disease. **(A)** MDA-MB-231 breast cancer cells were injected into the tail vein of Balb/C female nude mice. Mice (6 per group) were treated with UNC0642 (5 mg/kg) starting from the day after cell injection and continued for 10 weeks. Lungs have then been extracted, fixed and analysed through H&E. **(B)** Representative images of excised lungs analysed through H&E. Arrows indicate metastatic lesions. Squares correspond to blown up areas. Dot plot represents the number of metastasis identified per mouse. Bar graph shows the number of mice that were found to bear metastasis. **(C)** A second set of mice (6 per group) was treated starting from week 10, after proper establishment of the metastatic disease, for 4 weeks. At the end of the treatment, lungs were dissected, fixed and analysed by H&E staining. **(D)** Representative images of H&E staining of lungs. Arrows indicate metastatic lesions. Squares correspond to blown up areas. Dot plot represents the number of metastasis identified per mouse. Bar graph shows the number of mice that were found to have metastasis. Data are shown as mean ± SEM (unpaired, nonparametric Student's t-test, * p<0.05).

Previously, it has been shown that G9a KD is able to impact the formation and growth of metastases in lung, breast and head and neck cancer 12,37. Hence, we sought to determine whether treatment with a small molecule inhibitor against G9a could demonstrate similar anti-metastatic effects. In particular, the effects of UNC0642 treatment were investigated *in vivo* against both the ability of cancer cells to colonize the lungs and against metastatic tumor growth. The experimental lung metastasis assay was utilized through injecting MDA‑MB‑231 cells into the tail vein of Balb/C female nude mice. We first performed an experiment to determine whether G9a inhibition blocks colonization of breast cancer cells in the lung. Mice injected with MDA-MB-231 cells via the tail vein were treated with vehicle or UNC0642 (5 mg/kg) starting from the day after the cell injections (Figure 2A). Upon termination of treatment, lungs from mice were dissected and metastatic lesions were analyzed by Hematoxylin and Eosin (H&E) staining. G9a inhibitor treatment significantly reduced the number of metastases with no detectable macrometastases being found in 3 of the 5 mice treated with UNC0642 (Figure 2B).

We then sought to determine whether G9a inhibition is efficacious in treating established lung metastases. Mice injected with MDA-MB-231 cells were left untreated for 10 weeks to allow these cells to colonize and form tumors before treatment was administered. This was to test the efficacy of G9a inhibition in a fashion in which recapitulates the clinical setting of breast cancer recurrence. Tumor bearing mice were treated with vehicle or UNC0642 (5 mg/kg) for 4 weeks and lung tumors were analyzed (Figure 2C). Control mice presented with an elevated metastatic burden in the lung. In contrast, UNC0642 treated mice were characterized by a robust reduction in the number and size of metastatic lesions (Figure 2D). This data demonstrate for the first time that targeting G9a through the use of a small molecule inhibitor significantly blocks both metastasis formation and growth *in vivo*.

### G9a actively represses cell adhesion molecules during hypoxic stress

**Figure 3:**
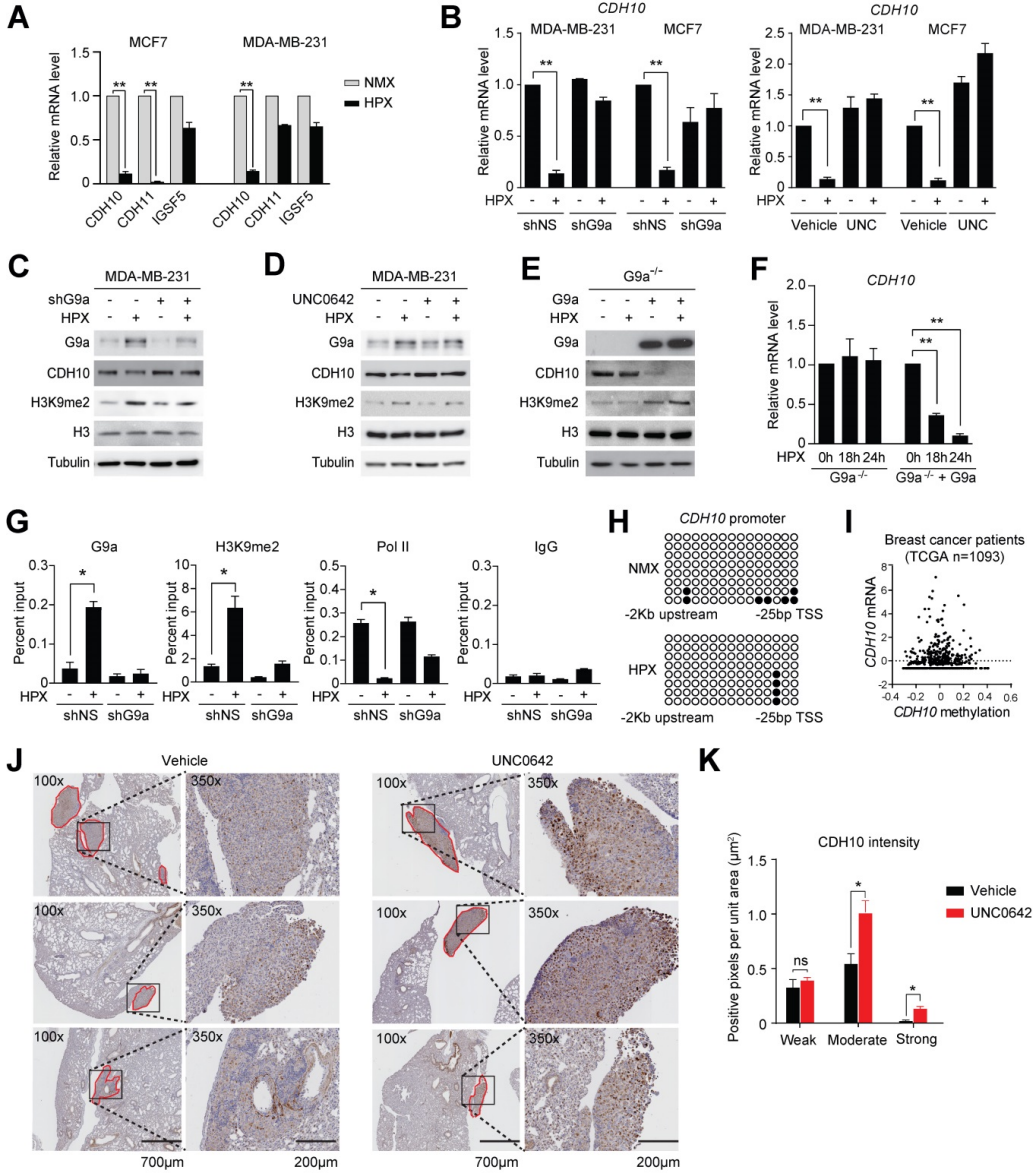
** G9a silences *CDH10* expression under hypoxic conditions.** G9a directly regulates the expression of the cell adhesion molecule CDH10 in breast cancer. **(A)** Quantitative PCR analysis of *CDH10*, *CDH11* and *IGSF5* expression in MCF7 and MDA‑MB-231 cells exposed to hypoxia for 9 hours. **(B)** Quantitative RT-PCR analysis of *CDH10* expression in MCF7 and MDA-MB-231 cells grown in normoxic and hypoxic conditions following G9a KD or UNC0642 (5 μM) treatment for 9 hours. **(C)** Western Blot analysis of CDH10 protein in MDA-MB-231 following G9a KD and **(D)** UNC0642 (5 μM) treatment, under normoxic and hypoxic conditions. H3 and Tubulin used as loading control. **(E)** Western Blot analysis of G9a, CDH10 and H3K9me2 in G9a^-/-^ MEFs reconstituted with WT G9a. Tubulin and H3 used as loading control. **(F)** *CDH10* mRNA expression evaluated at indicated time points in hypoxic conditions in G9a^-/-^ MEFs compared with the G9a reconstituted cells. **(G)** Chromatin Immunoprecipitation analysis of G9a, RNA polymerase II and H3K9me2 on *CDH10* promoter in MCF7 cells transfected with shNS or shG9a constructs exposed to normoxic and hypoxic conditions. **(H)** Bisulphite sequencing analysis of *CDH10* promoter in MCF7 cells grown in normoxic and hypoxic conditions. **(I)** CDH10 expression associated with DNA methylation levels in patient data (TCGA), showing no direct correlation between the two. **(J)** Representative images of immunohistochemistry analysis of CDH10 in lungs with established metastatic disease treated with or without UNC0642. **(K)** Intensity (number of positive pixel per unit area) of CDH10 expression in lung metastasis subdivided into low, positive and high positive. Data are represented as mean ± SEM of three independent experiments (unpaired, non-parametric Student's t-test, * p<0.05, ** p<0.005).

Loss of expression of cell adhesion molecules is believed to be the initial step in the promotion of cellular motility. Therefore, we investigated whether the observed induction in cellular motility following exposure to hypoxia may be dependent on the G9a-mediated repression of specific cell adhesion molecules. To do this, the G9a-dependent hypoxia repressed genes described earlier (ie. genes that were directly repressed by hypoxia but their expression was rescued upon G9a KD) were analyzed and cross-referenced with the HUGO database. Five genes known to encode for cell-adhesion molecules were identified (*CDH10*, *CDH11*, *CEACAM7*, *IGSF5* and *SIGLEC14*). In order to validate which of these genes affected cellular motility in both MDA-MB-231 and MCF7, cells were cultured in normoxic or hypoxic conditions for 9 hours and the expression of the five genes was analyzed by qPCR. Upon qPCR validation, it was determined that only 3 out of 5 genes were expressed in both MCF7 and MDA‑MB-231 (*CDH10*, *CDH11* and *IGSF5*). Out of these 3 genes, *CDH10* was the only gene robustly repressed by hypoxia in both cell lines (Figures 3A and S4A). In order to confirm that G9a is responsible for modulating *CDH10* expression, qPCR analysis was performed on RNA isolated from MDA-MB-231 and MCF7 cells grown in hypoxic or normoxic conditions following either G9a KD or treatment with UNC0642. Both G9a KD and UNC0642 treatment rescued hypoxia-mediated gene repression of *CDH10* (Figure 3B), where *IGFBP3* was used as a positive control for hypoxia (Figure S4B). Concomitantly, CDH10 protein levels were detected through Western immunoblotting under similar conditions and following exposure to hypoxia for 24 hours. While hypoxia was found to reduce CDH10 protein, G9a KD and UNC0642 treatment were able to block its hypoxia-mediated repression (Figures 3C, D and S4C). Because G9a was previously reported to modulate the expression of both E-cadherin (CDH1) and EpCAM, levels of the two proteins were analysed following exposure to hypoxia and treatment with UNC0642 (Figure S4D). Hypoxia led to a reduced amount of both proteins in MCF7, which was prevented by G9a inhibition. However, neither of the two proteins were observed in MDA-MB‑231. In particular, only CDH10 was present at detectable levels in the two cell lines, with MCF7 displaying higher levels than MDA-MB-231. To further demonstrate G9a-dependence in the regulation of CDH10, G9a-deficient (G9a^-/-^) mouse embryonic fibroblasts (MEF) exposed to hypoxic condition failed to show a reduction in *CDH10* transcript level, while introducing wild type (WT) G9a into these cells led to a robust reduction in *CDH10* mRNA and protein in hypoxia (Figures 3E and F).

To assess the importance of the methyltransferase activity of G9a in *CDH10* expression, G9a lacking its catalytic domain (SET domain) was overexpressed in G9a-deficient MEFs (Figures S4E and F). While overexpression of WT G9a led to a reduction in both CDH10 protein and mRNA, no effect was observed when G9a lacking SET domain was introduced. Because G9a is known to catalyze mono- and di-methylation of H3K9 for gene repression, Chromatin Immunoprecipitation (ChIP) analysis of the *CDH10* promoter was performed to investigate changes in H3K9 methylation in MCF7 cells exposed to hypoxic conditions following G9a KD (Figure 3G). Hypoxia increased G9a recruitment on the *CDH10* promoter, concomitantly with an increase in H3K9me2 levels, and reduced RNA Polymerase II (Pol II) recruitment, a marker of transcriptional activity. G9a depletion led to a robust reduction in H3K9me2 under both normoxic and hypoxic conditions, concomitantly with an increased recruitment of Pol II to the *CDH10* promoter in hypoxia, demonstrating that the *CDH10* promoter is under the direct control of G9a histone methylation in hypoxic conditions. In order to ascertain that the regulation of *CDH10* transcription is mediated by histone methylation in hypoxia and not DNA methylation, bisulfite sequencing of the *CDH10* promoter was performed (Figure 3H). No increase in DNA methylation was observed in hypoxic conditions compared to normoxic conditions. In addition, there was no correlation between *CDH10* promoter DNA methylation and the transcript level of *CDH10* in a breast cancer patient cohort from TCGA (Figure 3I). Finally, in order to understand whether G9a inhibition could regulate the expression of CDH10 *in vivo*, immunohistochemistry (IHC) analysis of CDH10 was performed on lung sections dissected from mice treated with UNC0642 following establishment of metastatic disease (Figures 3J and K). IHC demonstrated a significant increase in expression of CDH10 in metastatic lesions with UNC0642, demonstrating that inhibiting G9a is able to enhance CDH10 expression *in vivo*. Together, the data presented identified the type-II cadherin CDH10 as being transcriptionally regulated by G9a in hypoxia.

### CDH10 regulates cell motility in breast cancer cell lines

**Figure 4:**
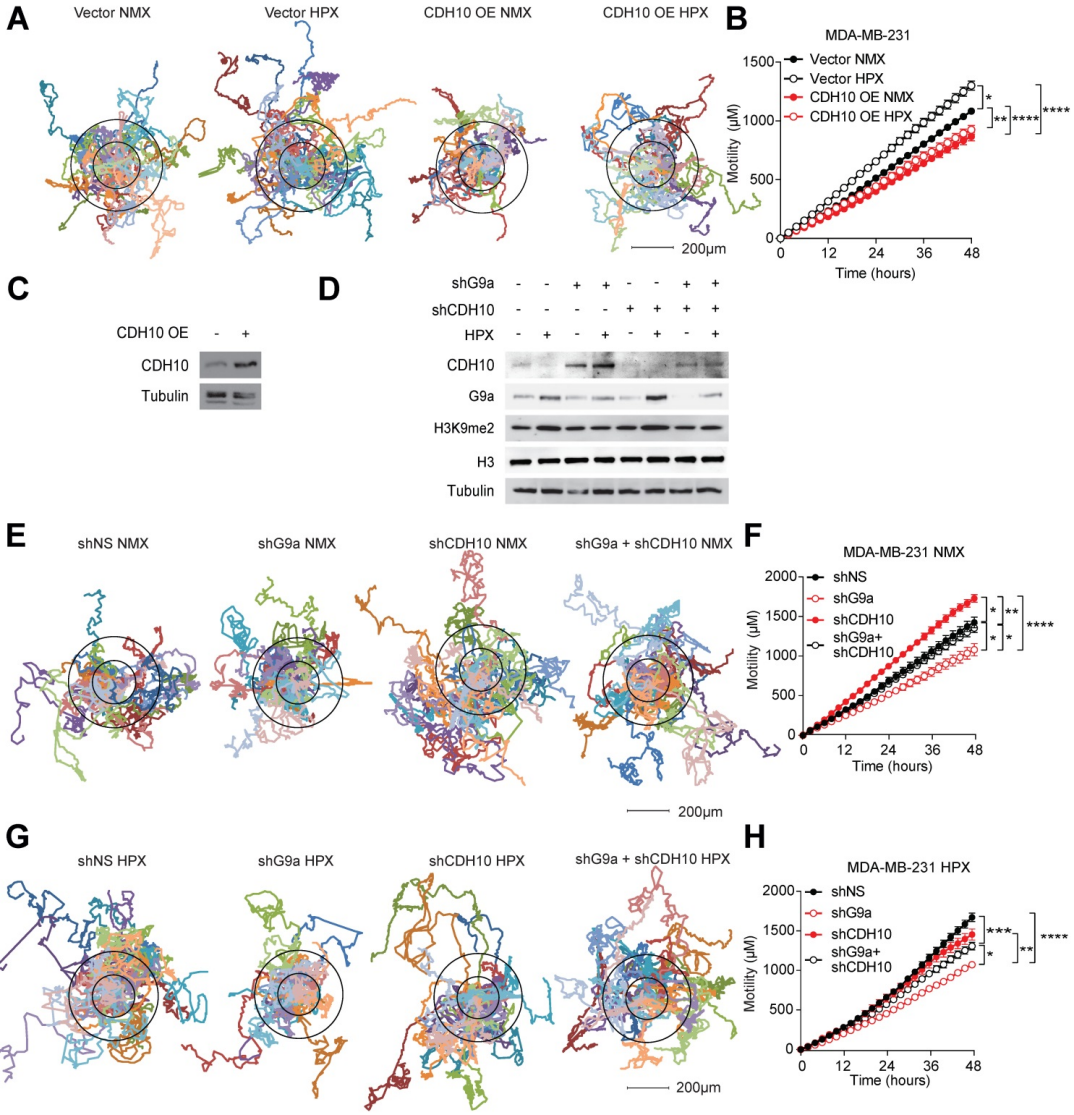
** CDH10 impacts cell motility in breast cancer.** CDH10 reduces cell motility in breast cancer cell lines. **(A)** Representative displacement plots of MDA-MB-231 cells tracked for 48 hours following CDH10 overexpression in normoxia and hypoxia. Shown is an average of 20 cells per condition. **(B)** Quantitation of cellular motility in CDH10 overexpressing cells under normoxic and hypoxic conditions. **(C)** Western immunoblotting analysis of CDH10 in MDA-MB-231 cells following transfection with empty vector or CDH10 overexpressing construct. **(D)** Western immunoblotting analysis of G9a and CDH10 under normoxia and hypoxia following transfection with shNS, shG9a, shCD10 or the double KD. **(E)** Representative displacement plots of MDA-MB-231 transfected with shNS, shG9a, shCDH10 or the double KD under normoxic conditions. Shown is an average of 20 cells per conditions. **(F)** Quantitation of cellular motility of shG9a, shCDH10 and double KD cells. **(G)** Representative displacement plots of MDA-MB-231 transfected with shNS, shG9a, shCDH10 or the double KD under hypoxic conditions. **(H)** Quantitation of cellular motility of G9a, CDH10 and double KD cells under hypoxia. Data are represented as mean ± SEM of three independent experiments (One-way ANOVA, * p<0.05, ** p<0.005, ****p<0.0001).

To assess whether changes in CDH10 protein levels are at least partially responsible for the motility phenotype observed in breast cancer cells in hypoxia, CDH10 was overexpressed in MDA-MB-231 cells and motility was examined in normoxic and hypoxic conditions. Hypoxia was able to increase motility of MDA-MB-231 cells transfected with the empty vector (Vector), while CDH10 overexpression (CDH10 OE) blocked the hypoxia-mediated increase in motility (Figures 4A-C). Moreover, CDH10 overexpression was also able to reduce cell motility even in normoxic condition, supporting the role of CDH10 as an adhesion molecule that can inhibit cancer cell motility phenotype. In addition, CDH10 KD in MCF7 cells resulted in an increased cellular motility, mimicking the effects observed during hypoxic stress (Figure S5). We then asked whether CDH10 played a major contribution in inhibiting motility following G9a KD. To do this, CDH10 and G9a double KD was performed in MDA-MB-231 (Figure 4D). Motility was then analysed under both normoxic (Figures 4E and F) and hypoxic conditions (Figures 4G and H). Under both conditions, blockade of CDH10 expression was able to counteract the ability of G9a depletion to reduce cellular motility. Collectively, these data support the possible role for CDH10 as an important factor modulating cell motility in breast cancer following exposure to hypoxia.

### *CDH10* as a prognostic marker in breast cancer patients

**Figure 5:**
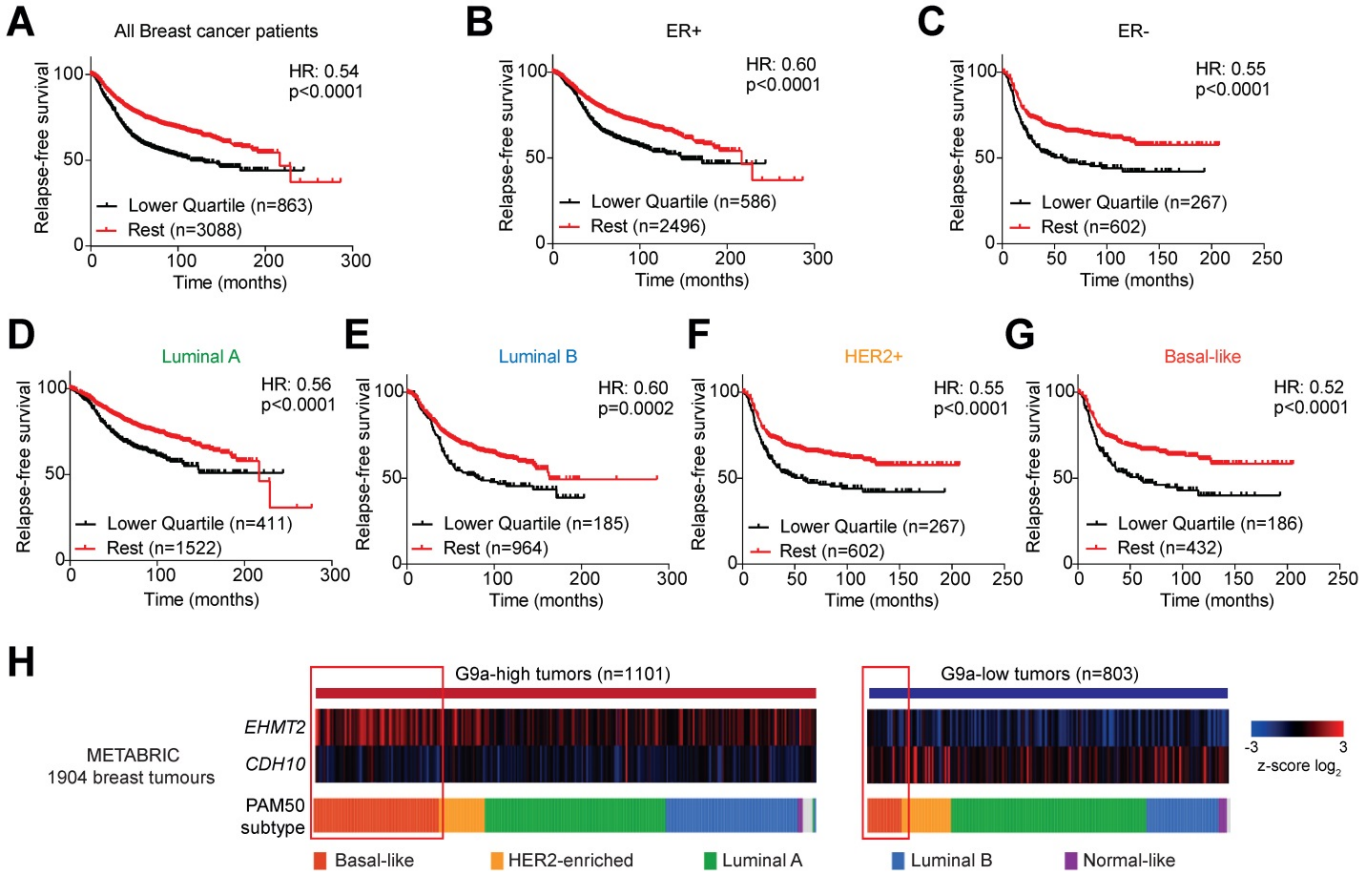
***CDH10* expression is prognostic in breast cancer.** *CDH10* expression is prognostic and inversely correlated to *EHMT2* expression in cancer. Kaplan-Meyer relapse-free survival analysis of *CDH10* expression in TCGA patient data in **(A)** all breast cancer patients, **(B)** ER+, **(C)** ER-, **(D)** Luminal A, **(E)** Luminal B, **(F)** HER2+ and **(G)** Basal-like. **(H)** METABRIC data analysis showing the level of expression of *CDH10* in correlation with *EHMT2* levels in tumor samples divided into different subtypes. Red squares highlight for both groups the proportion of patients belonging to the basal-like subtype.

Publicly available databases were examined to evaluate the importance of G9a (*EHMT2*) and *CDH10* expression in breast cancer. The ability of *EHMT2* and *CDH10* mRNA to stratify breast cancer patients was analysed in the KM Plotter database. *EHMT2* transcript levels were found not to be a good marker for relapse-free survival in breast cancer patients. In particular, no stratification was observed examining all breast tumors (Figure S6A), or subdividing the samples into ER+ and ER- (Figures S6B and C) and molecular subtypes (Figures S6D-G). The sole exception was Luminal B tumors, where high *EHMT2* was significantly associated with a worse relapse-free survival (Figure S6E). As hypoxia stabilises G9a protein levels, it is possible that the evaluation of the expression of G9a target genes provides a better representation of G9a activity in patient samples. *CDH10* transcript levels were therefore examined in breast cancer patients and it was found that higher *CDH10* expression was associated with improved relapse-free survival (Figure 5A). Further dividing patients into ER+ and ER- tumors revealed that *CDH10* was significantly correlated with better prognosis in both subtypes, but it performed better in the ER- cohort, as demonstrated by the lower hazard ratio (HR) (Figures 5B and C). ER- tumors are generally more aggressive than ER+, characterized by an increased risk for metastasis. In this context, the prognostic value of *CDH10* further strengthens the idea of a protective role against metastasis formation. The prognostic power of *CDH10* was further evaluated by investigating its association with relapse-free survival in the different breast cancer molecular subtypes, or intrinsic subtypes, Luminal A and Luminal B for the ER+ tumors and HER2+ and basal-like for the ER- tumors (Figures 5D-G). While *CDH10* expression was prognostic in all the breast cancer subtypes analysed, the ER- subtypes HER2+ and basal-like were characterized by a stronger correlation (Figures 5F and G). In particular, the highly metastatic basal-like subtype displayed the best correlation, with the lowest hazard ratio between all the observed subtypes. Data presented in this part of our study suggest that *CDH10* expression is therefore prognostic in breast cancer, notably in more aggressive ER- subtypes.

Given the negative correlation described between G9a and CDH10, low *CDH10* expression in tumors would be expected to most commonly associate with increased *EHMT2* expression. To confirm this in breast cancer patients, the METABRIC database was used to evaluate the level of correlation between the mRNA expressions of *CDH10* and *EHMT2* (Figure 5H). Subdividing patients into *EHMT2* high and low tumors, a clear negative correlation between *EHMT2* and *CDH10* expression was observed. *EHMT2* high tumors were generally associated with a reduced *CDH10* expression, while *EHMT2* low tumors associated with high *CDH10* mRNA levels. By dividing the samples by subtype, basal-like tumors were found to express higher *EHMT2* transcripts more frequently compared to other molecular subtypes. Overall, our patient data analysis supports the *in vitro* finding of a negative correlation between G9a and CDH10 expression at both the mRNA and protein levels.

### G9a is frequently upregulated in various cancer types and associates with loss of *CDH10* expression

**Figure 6:**
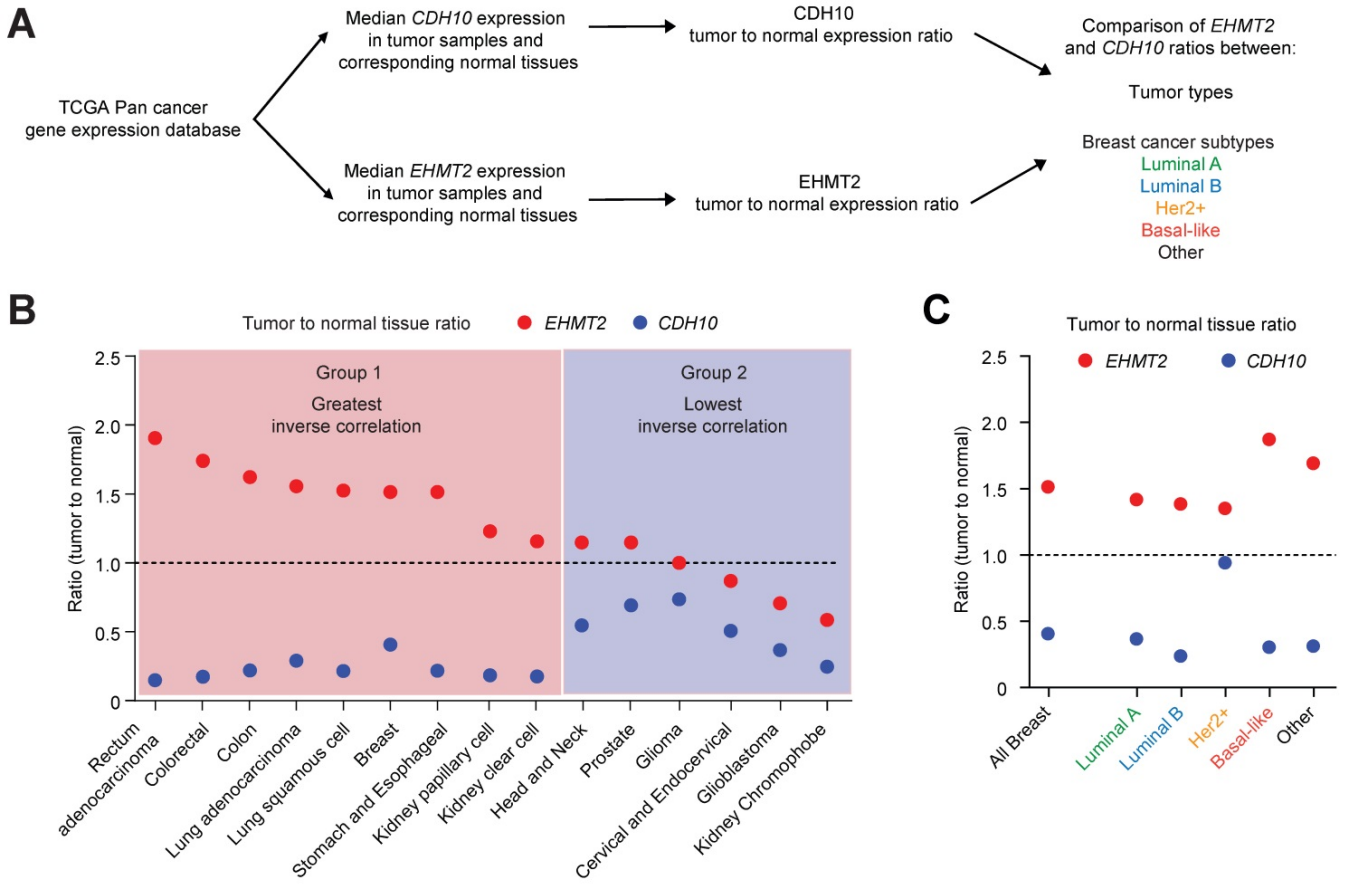
***CDH10* expression is frequently lost and inversely correlated to *EHMT2* in various cancer types.** *CDH10* expression is frequently reduced in cancer, when compared to normal tissues. **(A)** Flow chart describing the analysis performed on TCGA patient gene expression data, comparing *EHMT2* and *CDH10* expression level between tumor and normal samples in various cancer types and in the different breast cancer molecular subtypes. **(B)** Ratio of median expression of *CDH10* and *EHMT2* in tumor samples versus normal tissues in various cancer types (TCGA data). **(C)** Ratio of median expression of *CDH10* and *EHMT2* in tumor samples versus normal tissues in the different breast cancer molecular subtypes (TCGA data).

Mutations and loss of expression of *CDH10* are associated with various cancers types 30,31,38. However, when the mutational status was investigated in breast cancer patients using publicly available databases (through cBioportal), no significant frequency of gene alterations was identified for both *EHMT2* and *CDH10* (Figure S7A). This is in contrast to lung cancer which was previously reported to be frequently associated with mutations on the *CDH10* gene, where a significantly higher percentage of *CDH10* alterations were detected (Figure S7B). We therefore investigated whether loss of *CDH10* was common in breast cancer and other tumor types and whether this was associated with an increased *EHMT2* expression. Taking advantage of TCGA pan-cancer database, *EHMT2* and *CDH10* expression levels were obtained in cancers for which gene expression data was available for both normal and tumor tissues. Median expression of *EHMT2* and *CDH10* in tumors was compared with their respective expression in normal tissues and the two ratios were then compared with each other in each cancer type (Figure 6A and Table S2). The majority of tumor types analysed displayed a loss of expression of *CDH10* when compared to normal tissue expression levels. In contrast, *EHMT2* was frequently upregulated in tumor samples (Figures S8A and B). When the two ratios were compared with each other and tumors were ordered from the highest *EHMT2:CDH10* ratio to the lowest, 9 out of 15 cancer types were characterised by a strong inverse correlation between the two genes (Figure 6B). In these tumors, *EHMT2* expression was elevated when compared to normal tissues, while *CDH10* expression was concomitantly lost (group 1: rectum, colorectal, colon, lung, breast, stomach and esophageal, kidney papillary and clear cell). In the other 6 cancer types (group 2: head and neck, prostate, glioma, cervical, glioblastoma and kidney chromophobe) *CDH10* expression was lost or reduced, but this was not correlated with increased *EHMT2* expression. The breast cancer samples were then further stratified into the different molecular subtypes and the expression ratios of tumor to normal tissue for *CDH10* and *EHMT2* were compared as previously (Figures 6C and S9A-F). It was clear that *EHMT2* expression was higher in all subtypes when compared to normal tissues, and was associated with a loss of *CDH10*. Interestingly, the HER2+ subtype constituted the only exception, with *CDH10* expression being comparable to the normal tissue. In contrast, basal-like tumors were characterized by the highest levels of *EHMT2* transcripts and by the greatest inverse correlation with *CDH10*. In addition, the correlation between G9a and CDH10 protein was investigated using a publicly available database of 65 breast tumors and 53 adjacent non‑cancerous tissues analysed through quantitative liquid chromatography /mass spectrometry-based proteome analysis (Figure S9G) 39. In the database, a number of patients did not display detectable protein levels of G9a (n=18) and CDH10 (n=21). In patients where expression of both proteins was identified (n=26), a negative correlation between CDH10 and G9a protein levels was observed.

Collectively, our results demonstrate that inhibiting G9a activity reduces cellular motility and metastatic spread in breast cancer and promotes *CDH10* expression under hypoxic conditions. Consistent with this, expression of *EHMT2* and *CDH10* are inversely correlated in multiple cancer types. CDH10 reduces cancer cellular motility and loss of the protein may represent an important step in the hypoxia-mediated modulation of cellular motility. Furthermore, *CDH10* is a prognostic biomarker in breast cancer, especially in ER- subtype. We propose that CDH10 possesses a metastasis suppressive function in breast cancer and that G9a represents an attractive target for the treatment of hypoxia-driven metastatic breast cancer.

## Discussion

Hypoxia is a critical factor in solid tumors, known to associate with a more aggressive phenotype and to increase the risk of metastasis 2,3,40. For this reason, the inhibition of hypoxic signaling in neoplastic lesions is believed to be highly beneficial for cancer patients. In the cellular response to environmental conditions, epigenetic mechanisms are known to play an important role in modulating gene expression to adapt and promote cancer cell survival 10.

During the development of invasive metastatic cancer, neoplastic cells undergo profound changes that alter their ability to interact with the extracellular matrix, disrupting cell adhesion and apical-basal polarity, promoting cellular motility and invasiveness. This mechanism is a hallmark of the EMT process, where cells lose an epithelial phenotype and acquire mesenchymal characteristics 41,42. In particular, when EMT is induced, cells undergo a process referred to as “cadherin switch” where the expression of specific epithelial cadherins are lost (such as E-cadherin) and mesenchymal are re-expressed (N-cadherin), leading to a more metastatic behaviour in cancer 43.

G9a oncogenic role has been reported in various types of cancer, such as ovarian, lung and breast 44. We previously described that G9a protein increases during hypoxia, leading to the modulation of specific hypoxia-responsive genes. Here, we demonstrate that hypoxic stress leads to enhanced motility of breast cancer cells which can be attenuated by inhibition of G9a. We also demonstrate that G9a is required for various steps of the metastatic cascade in breast cancer, including cellular motility, lung colonisation and metastatic growth, which supports the notion of G9a as an oncogene. Similar results have also been obtained by others in various cancer types, such as lung and gastric cancer. In particular, G9a overexpression was shown to increase lung metastasis and enhance the formation of disseminated tumors in gastric cancer 12,45. Another study reported that G9a knockdown repressed growth and lung colonisation of breast cancer cell lines 46. Our data confirm these results and demonstrate for the first time that metastasis formation and growth could be abrogated using a small molecule inhibitor against G9a methyltransferase. This approach is more clinically relevant as knock down of G9a protein is not an ideal approach in humans and suggests the feasibility of targeting G9a to block metastasis in cancer patients. While a similar result was previously reported using a dual inhibitor against G9a and DNMT1, our work confirms that inhibiting G9a is sufficient to block metastasis formation 47. However, there is also evidence for a tumor suppressor role for G9a in specific cancer contexts where the enzyme suppresses the tumor-propagating cell phenotype in aggressive lung cancer 48. In our study, G9a inhibitor treatment blocked metastasis *in vivo* without the propagation of more aggressive breast cancer cells.

It has previously been shown that G9a is able to control the expression of E-cadherin by histone methylation, binding to the transcription factor SNAIL that mediates its recruitment to the gene promoter 46,49. The EpCAM was also found to be repressed by G9a in lung cancer 50. Our data demonstrate that G9a can also regulate the expression of another cell adhesion molecule, namely CDH10, which may be important in the modulation of cellular motility in response to hypoxia. CDH10 expression was not associated with any change in the levels of DNA methylation at the promoter level, suggesting that G9a directly regulates *CDH10* expression through histone methylation. We also showed that CDH10 is able to reduce motility of breast cancer cells and attenuate the hypoxic response, suggesting that, at least partially, loss of CDH10 is responsible for the increased migratory potential observed during hypoxic stress.

CDH10 is a type II cadherin belonging to the cell to cell adhesion molecule family. Following oxygen depletion in the primary tumor, EMT activation and subsequent loss of expression of CAMs is considered to be an important initial factor in the metastatic cascade 51. Because of the association between metastasis and poor survival rates in cancer patients, understanding the key players regulating the process is fundamental for the development of new targeted therapies, as well as for an accurate prediction of long-term outcome in cancer patients 6. We found that *CDH10* is prognostic in breast cancer patients, with greater influence on the ER- subtype. This subtype is generally characterized by poor survival rates, mainly due to the lack of proper targeted therapies and the frequent onset of metastasis. Interestingly, the more aggressive ER- subtype, the basal-like, displayed the best correlation between *CDH10* expression and relapse-free survival and was also correlated with the highest *EHMT2* expression levels. These data suggest that CDH10 may play a protective role against metastasis formation in aggressive breast tumors. CDH10 could therefore represent a biomarker for patient stratification, allowing the early detection of tumors with the highest risk of developing distant metastasis and poor outcomes.

A number of recent studies have described a correlation between the loss of *CDH10* expression and cancer. CDH10 has been proposed to act as a tumor suppressor in lung cancer, where its overexpression was shown to impact both the proliferative and motility potential of tumor cells 38. Mutations in *CDH10* that lead to a loss of expression are associated with pancreatic ductal adenocarcinoma and metastatic small-cell gallbladder neuroendocrine carcinoma 30,32. It has been also identified as being recurrently mutated in colorectal cancer 52. Moreover, *CDH10* was described to be prognostic in prostate cancer and its expression was found to be lost in aggressive forms of the disease 31,53. These results, combined with our findings, suggest that CDH10 might have a protective role against metastasis in various cancer types and that its expression is frequently lost in aggressive forms of the disease. Apart from breast cancer, we demonstrated by pan-cancer analysis of TCGA patient expression database that loss of *CDH10* was common in other cancer types. In particular, in 9 out of 15 cancer types analysed, loss of *CDH10* was associated with an increased expression of *EHMT2*. This association is consistent with the ability of G9a to repress *CDH10* transcription. It is interesting to observe that cancers originating within similar areas of the body tended to cluster in the same group. For example, group one is highly enriched in tumors originating in the gastrointestinal tract and the lung. In addition, kidney cancers clustered to the right end of groups 1 and 2, which for both groups corresponded to the lowest expression of *EHMT2*, as well as low *CDH10* expression. Kidney cancers are known for being characterized by dysregulations of hypoxic signaling. The majority of these tumors lack *VHL* expression, leading to constitutive G9a protein accumulation 10,54. For this reason, in this cancer type, *EHMT2* levels may not reflect its actual regulatory activity. In accordance with this model, *CDH10* transcript levels in kidney cancers were found to be strongly reduced regardless of *EHMT2* expression. Consistent with this knowledge, an independent study also identified *CDH10* to be significantly underexpressed in kidney tumor samples lacking pVHL 54.

In conclusion, we have demonstrated that inhibiting G9a can block breast cancer cell motility by directly re-expressing *CDH10.* We propose that CDH10 loss is an important step in the hypoxia-mediated EMT process in breast cancer. We also propose that CDH10 possesses metastasis suppressor function and that G9a represents an attractive target for the treatment of metastatic breast cancer.

## Materials and Methods

### Cell culture

Human breast cancer cell lines were obtained from ATCC and cultured as per ATCC instructions. All cell lines were regularly tested for mycoplasma and authenticated using short tandem repeat profiling. For hypoxic experiments, cells were incubated in a hypoxia workstation (Ruskinn InVivo 1000 Dual chamber) set at 1% O_2_ and 5% CO_2_. Prior to the start of the experiment, media was refreshed with full media pre-conditioned in hypoxia for at least 24 hours.

### Holographic imaging and cell motility

Cell motility was analysed by the holographic microscope HoloMonitor M4 (Phase Holographic Imaging, Lund, Sweden). Cells (5×10^3^/well) were seeded in a six well plate and allowed to attach for 24 h prior to treatment with either G9a inhibitor (UNC0642) or vehicle (DMSO). Digital images were captured every 10 minutes for 96 and 48 hours and cell motility was analysed using Hstudio M4 2.6.3 (Phase Holographic Imaging, Lund, Sweden). The distance covered over the 48 hours has been determined for a total of 20 individual cells per treatment, merged from three independent experiments. For the displacement graphs, boxed position (X and Y) were used to plot cellular movements in 2D.

### IncuCyte real-time imaging

Cell motility assays were performed plating MCF7 and MDA MB-231 cells at a number of 2 x 10^4^ and 2.5 x 10^4^ per well respectively, in 96 wells plates. When the cells reached confluence, a scratch was performed at the centre of the well using the Wound Maker (Essen BioScience). Images were acquired every 6 hours. For invasion assays, 96 place-well plates were coated with matrigel (Corning, 100 μg/ml) overnight. Cells were then seeded and a scratch was made. Matrigel (8 mg/ml) was then added and allowed to solidify for 30 minutes at 37°C prior to starting image capture.

### Bisulphite sequencing

Genomic DNA (500 ng) was chemically deaminated by sodium bisulphite to convert unmethylated cytosines to uracils, leaving methylated cytosines unaltered, using the EZ DNA Methylation Kit Gold (Zymo Research) according to the manufacturer's instructions. Promoters were then amplified by PCR using primers designed using MethPrimer. Sequencing results were analyzed using BiQ Analyzer.

### Western immunoblotting

Protein assays were performed according to the Bradford method using a Bio-Rad protein assay kit (Bio-Rad). Denatured proteins were separated using sodium dodecyl sulfate-polyacrylamide gel electrophoresis (SDS-PAGE) and then transferred to PVDF membranes. The membranes were blocked in 5% skimmed milk in Tris-buffered saline with Tween 20 (TBST; 10 mM Tris-HCl, 150 mM NaCl, 0.1% Tween 20). Immunoblotting was performed with a number of different primary antibodies at optimal dilutions (see antibodies). The membranes were incubated overnight at 4 °C, rinsed with TBST, and incubated with horseradish peroxidase-conjugated anti-rabbit secondary antibody (Cell Signaling Technology). After applying ECL detection reagents (GE Healthcare), protein bands were visualized using X-ray film (Fujifilm).

### Antibodies

The following commercially available antibodies were used: CDH10 (Sigma, HPA010651, also used for Immunohistochemistry), HIF1α (Novus Biologicals, NB100-479), G9a (Abcam, 07-551), Tubulin (Sapphire Biosciences, STJ96939), Lamin A/C (Upstate, 05-714), GFP (Santa Cruz, SC-9996), H3K9me2 (Abcam, AB32521) and H3 (Abcam, AB1791).

### Microarray Statistical Analysis and Identification of Differentially Expressed Genes (DEGs)

Briefly, MCF7 cells transfected with non-silencing control shRNA or shRNA targeting G9a were exposed to normoxia or hypoxia for 9 h, and total RNA was isolated with the RNAeasy Mini Kit (Qiagen); 500 ng of total RNA was used for microarray analysis. The microarray analysis was performed using the Affymetrix Human Gene 1.0 ST Array. Dataset is deposited under GEO accession number GSE59449.

### *In vivo* xenograft studies

Balb/C nude female mice (6 per group), 6-8 weeks old, were injected with 5 x 10^5^ MDA-MB-231 cells through the tail vein. In the first experiment, mice were treated with 5 mg/kg UNC0642 (Sigma) IP every two days starting from the day after the cell injection. Treatment continued for 10 weeks and lungs were then extracted, fixed and analysed through H&E staining. In the second experiment, treatment started after 10 weeks from the cell injection, and continued for 4 weeks as described for experiment one. Lungs were then extracted and stained with H&E.

### Transfections

Transfections were performed using Lipofectamine 3000. Lentiviral transfections with viral packaging vectors (pNHP and pVSVG) were transfected into HEK293T cells. shRNA mediated knockdown plasmids against G9a were generated as previously described 55.

### Quantitative Real-Time RT-PCR and ChIP Assays

Quantitative RT-PCR and ChIP assays were conducted as previously described 10. Briefly, cells were fixed with 1% formaldehyde prior harvesting in collection buffer (100 mM Tris pH 9.4 and 10 mM dithiothreitol) and incubated at 30 °C for 15 minutes. Cells were then sequentially washed in buffer I (10 mM EDTA pH 8, 0.5 mM EGTA pH 8, 10 mM HEPES pH 6.5 and 0.25% TritonX-100) and Buffer II (1 mM EDTA pH 8, 0.5 mM EGTA pH 8, 10 mM HEPES pH 6.5 and 200 mM NaCl) and subsequently lysed in lysis buffer (10 mM EDTA pH 8, 50 mM Tris pH8.1, 1% SDS and protease inhibitor). Samples were then sonicated six times for 10 seconds and diluted in dilution buffer (2 mM EDTA pH 8, 20 mM Tris pH 8.1, 150 mM NaCl, 1% Triton X-100 and protease inhibitor). They were subsequently incubated with 2 μg of target antibody and magnetic beads (Millipore) at 4 °C overnight. Samples were then washed six times with wash buffer (1 mM EDTA pH 8, 50 mM HEPES pH 7.6, 0.5 mM LiCl, 1% NP-40 and 0.7% sodium deoxycholate). Chromatin was then eluted in elution buffer (100 mM NaHCO3 and 1% SDS) and cross-link was reversed adding 200 mM NaCl and incubating at 65 °C overnight. Gel Extraction Kit (Qiagen) was used to purify chromatin and samples were then analysed by qPCR with designed to be within 500 bp from the promoter region of the gene. For qPCR analysis, total RNA was isolated using Trizol (Invitrogen) and reverse transcription was performed from 2.5 μg of total RNA using the Superscript III cDNA synthesis kit (Invitrogen). The abundance of mRNA was detected using the ABI VIIA7 system with SYBR Green Master Mix (Life Technologies). Primer pairs were designed to amplify 90-150 bp mRNA specific fragments and were confirmed as unique products by melting curve analysis. The quantity of mRNA was calculated using the ΔΔCt method and normalized by using primers to detect *HPRT*. All reactions were performed in triplicates. See Table S1 for primer sequences used.

### Statistical Analysis

Statistical differences in test and control samples were determined by Student's t-test (Mann-Whitney) or ANOVA (Kruskal-Wallis) for group comparisons. Statistical analyses are performed using GraphPad Prism software.

## Supplementary Material

Supplementary figures and tables.

## Abbreviations

CDH1: Cadherin 1/e-cadherin
CDH10: Cadherin 10
ChIP: Chromatin Immunoprecipitation
DEG: Differentially expressed gene
EHMT2: Euchromatic histone methyltransferase 2
EMT: Epithelial to mesenchymal transition
EpCAM: Epithelial cell adhesion molecule
H&E: Haemotoxylin and Eosin
IP: Intraperitoneal
IPA: Ingenuity Pathway Analysis
TNBC: Triple negative breast cancer
pVHL: Von Hippel-Lindau
