# Supplementary material for: G9a-mediated repression of CDH10 in hypoxia enhances breast tumour cell motility and associates with poor survival outcome
Source: Theranostics. 2020 Mar 15;10(10):4515–29. doi: 10.7150/thno.41453 (PMC7150496; doi:10.7150/thno.41453)
Supplement: Supplementary file 1 — Supplementary figures and tables. [file thnov10p4515s1.pdf]

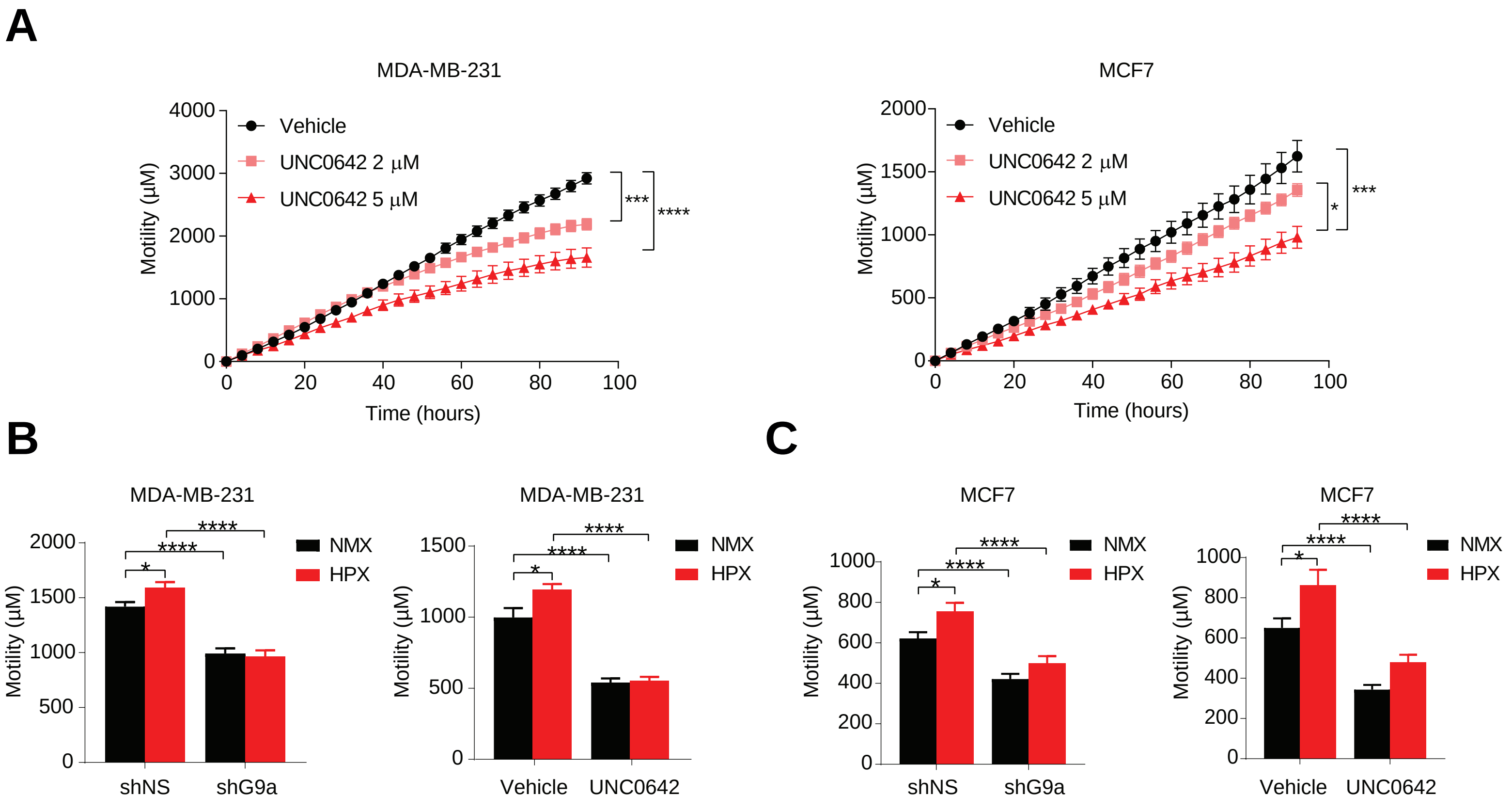

Figure S1
(A) Holomonitor analysis of cellular motility in MCF7 and MDA-MB-231 cell lines following treatment with UNC0642 (2 or 5 μM) for 96 hours. (B) Bar graph representing the total distance covered by MDA-MB-231 and (C) MCF7 tracked through holographic microscopy for 48 hours under normoxic (21% O_2) or hypoxic (1% O_2) conditions, following G9a knock down or UNC0642 treatment (5 μM). Data are represented as mean ± SEM (One-way ANOVA, * p<0.05, *** p<0.0005, **** p<0.0001).

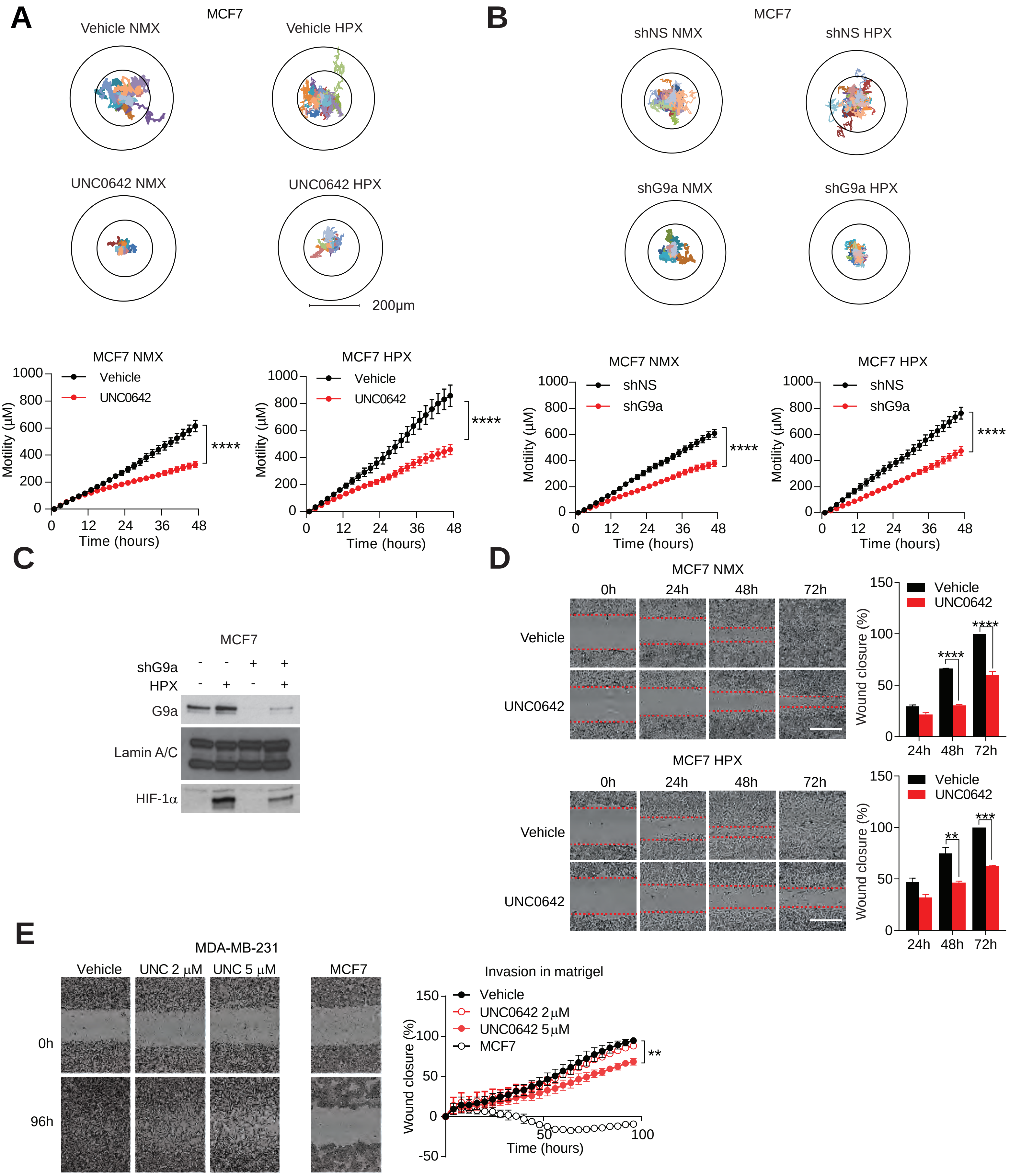

Figure S2. (A) Evaluation of the migratory distance covered by MCF7 cells treated with or without 5 µM UNC0642 for 48 hours or (B) following G9a KD. Results were evaluated through real-time imaging using the Holomonitor M4, taking pictures every 10 minutes. An average of 20 cells per condition is shown for the representative displacement images. (C) Western Blot analysis of G9a in MCF7 following G9a KD. (D) Scratch wound assay of MCF7 breast cancer cells treated with UNC0642 (5 µM) under both normoxic (21% O_2) and hypoxic (1% O_2) conditions. (E) Matrigel invasion assay of MDA-MB-231 and MCF7 cells in the presence of indicated concentrations of UNC0642. Graph summarises percentage of wound closure at every imaged time. Results were evaluated by real-time imaging performed by the IncuCyte Zoom every 24 hours and wound closure was quantified using ImageJ. Data are represented as mean ± SEM (non-parametric, Student t-test),*$p<0.05$, ** $p<0.005$, **** $p<0.0001$.

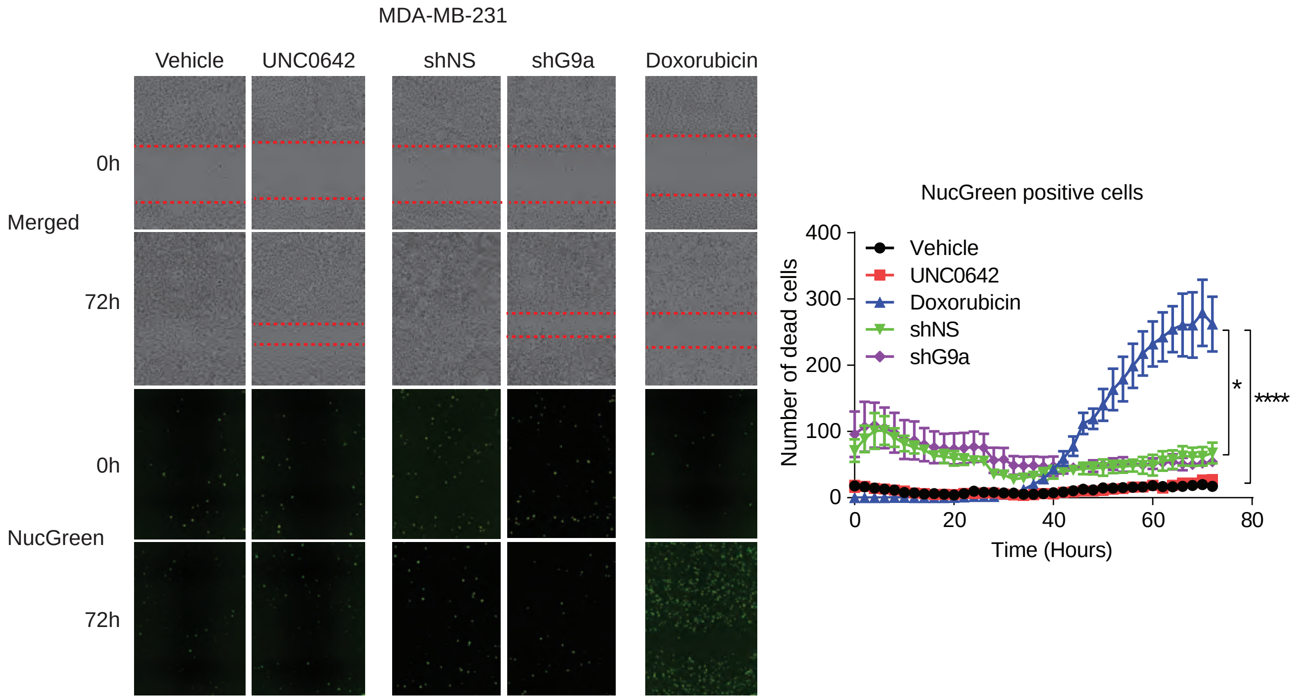

Figure S3
Scratch wound healing assay in MDA-MB-231 cells treated with UNC0642 (5 μM) or following G9a KD in the presence of NucGreen dead cells stain. Top phase-contrast images are merged with Green channel. Bottom images show only the relative green signal. Graph represents NucGreen quantification over time. Doxorubicin (1 μM) was used as positive control. Data are represented as mean ± SEM (non-parametric, Student's t-test), * $p<0.05$, **** $p<0.0001$).

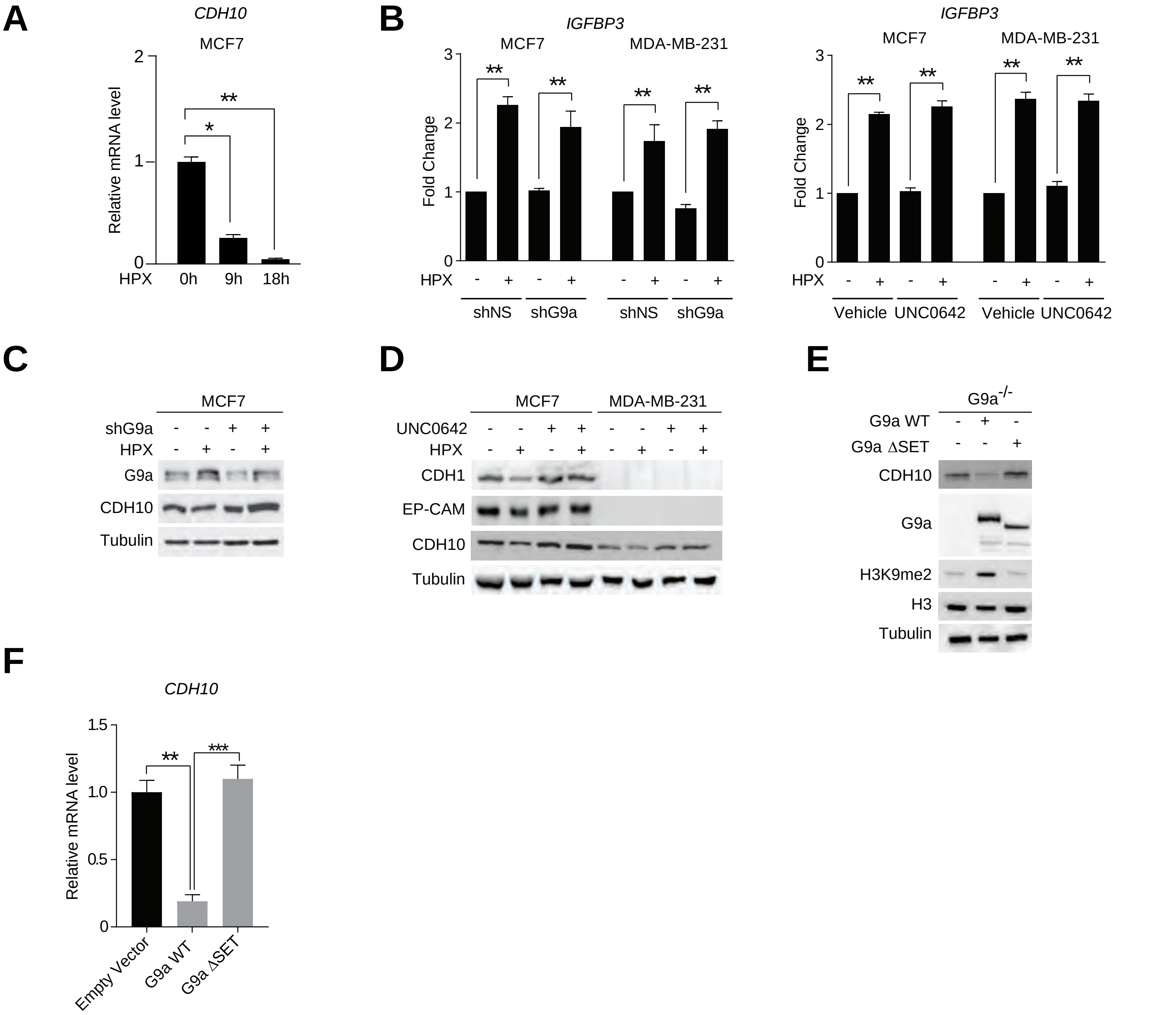

Figure S4. (A) CDH10 expression in MCF7 cells cultured under hypoxic conditions for the indicated times. (B) *IGFBP3* expression evaluated in MDA-MB-231 and MCF7 cells as positive control for the hypoxic environment. (C) Western Immunoblotting analysis of CDH10 in MCF7 cells transfected with shG9a and exposed to normoxia or hypoxia for 24 hours. (D) Western Immunoblotting analysis of CDH1, EpCAM and CDH10 in MCF7 and MDA-MB-231 following exposure to normoxia or hypoxia for 24 hours in the presence or in the absence of UNC0642 (5 μM). (E) Western immunoblotting analysis of CDH10 and H3K9me2 in G9a$^{-/-}$ MEFs transfected with WT G9a or G9a ΔSET. (F) *CDH10* mRNA levels in G9a$^{-/-}$ MEFs transfected as described. Data are represented as mean ± SEM (non-parametric, Student's t-test), * $p<0.05$, ** $p<0.005$, *** $p<0.005$.

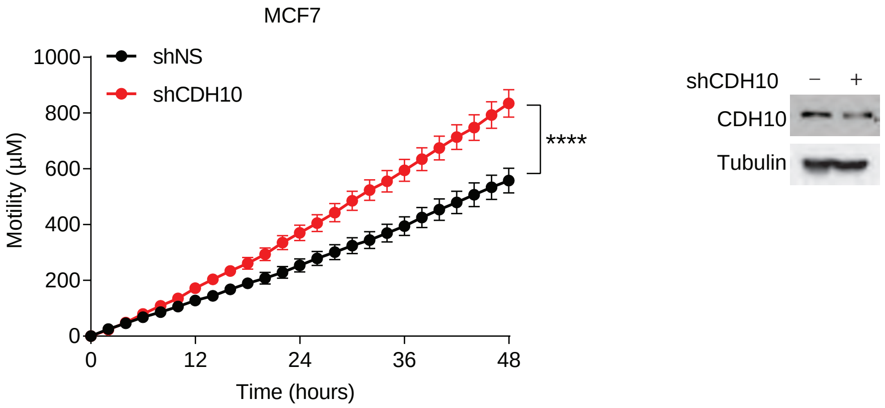

Figure S5. Evaluation of the migratory distance covered in 48 hours by MCF7 following CDH10 KD using the HoloMonitor M4. Data are represented as mean ± SEM (non-parametric, Student's t-test), **** p<0.0001.

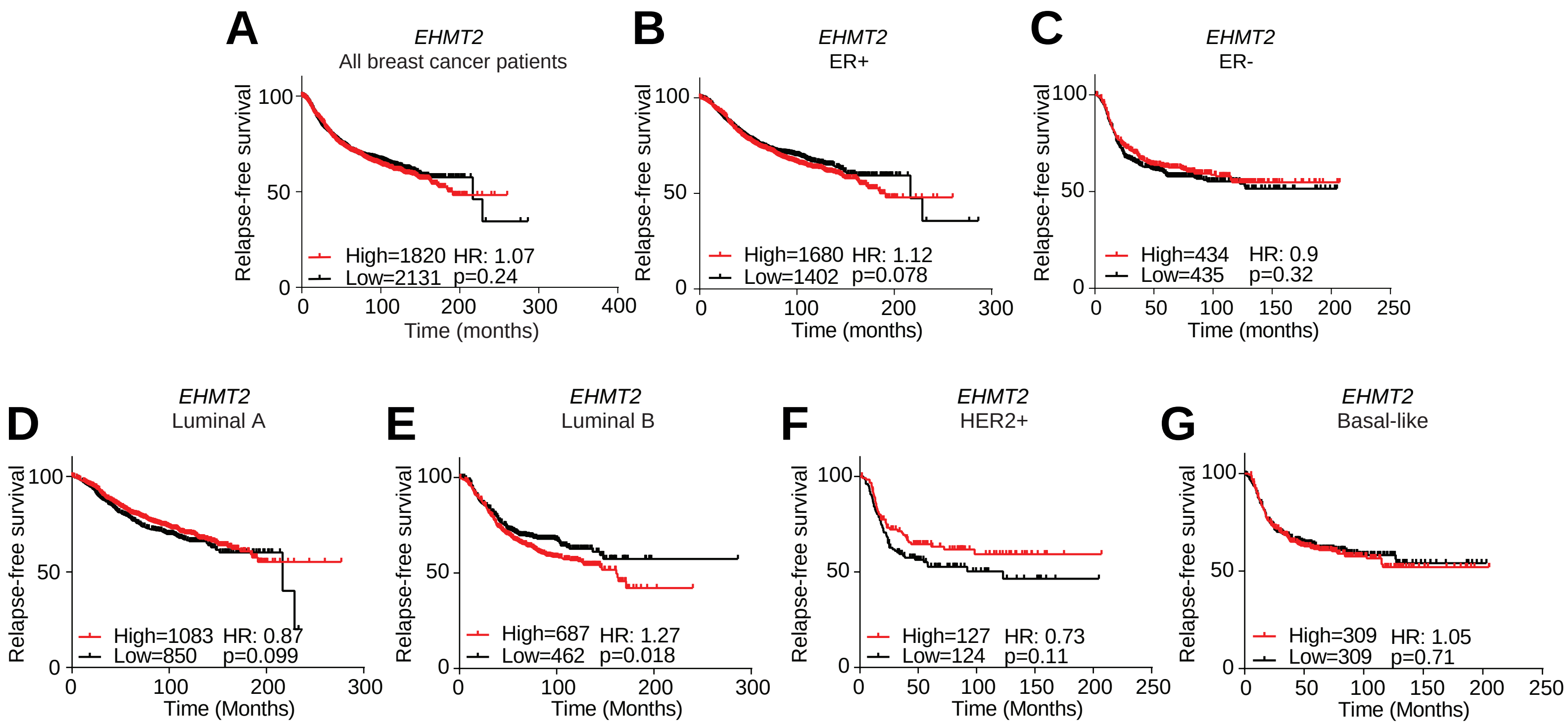

Figure S6. Kaplan-Meyer relapse-free survival analysis of EHMT2 expression in (A) all breast cancer patients, (B) ER+, (C) ER-, (D) luminal A, (E) luminal B, (F) HER2+ and (G) basal-like.

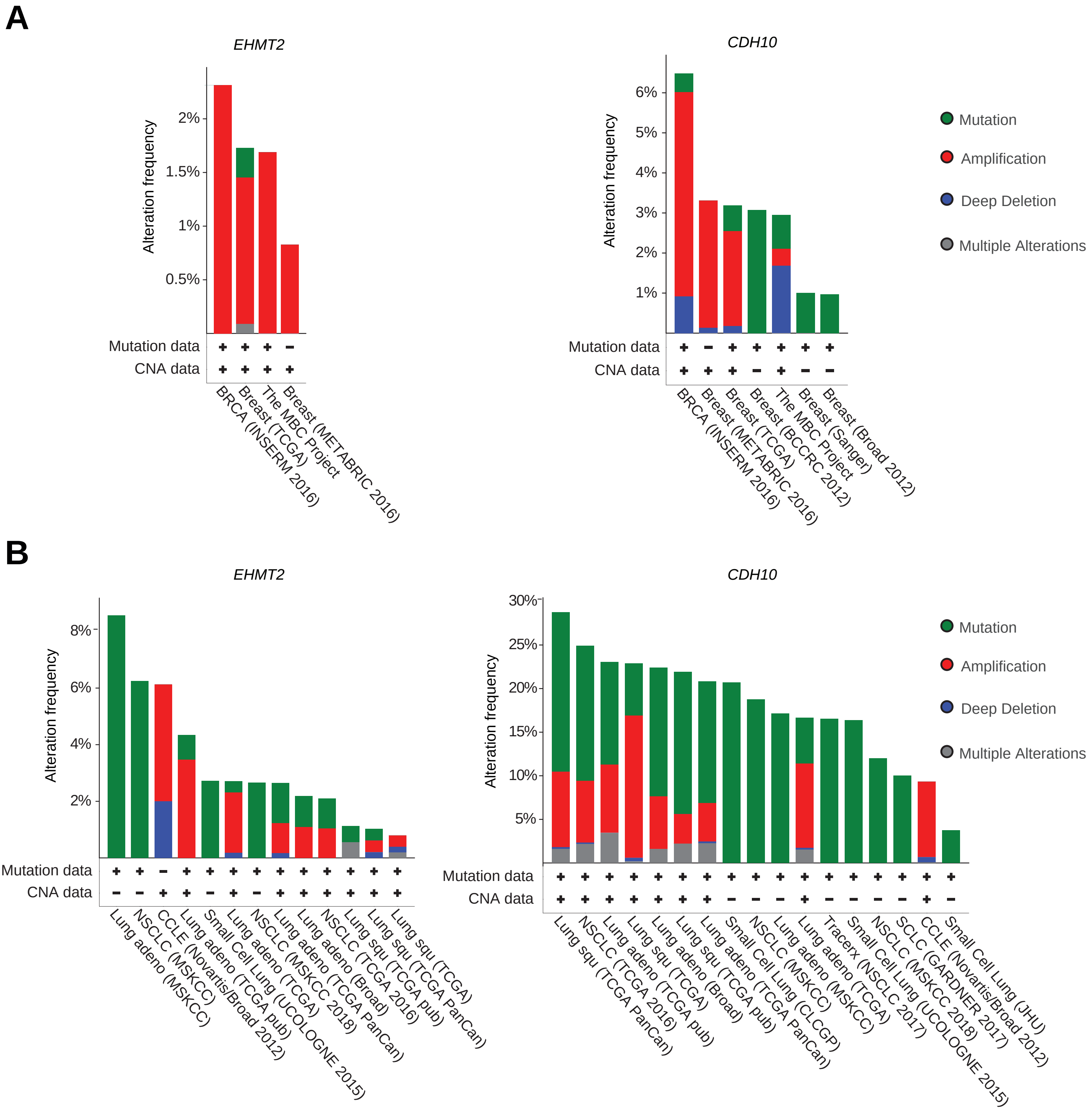

Figure S7. (A) Percentage and type of alterations in the *EHMT2* and *CDH10* genes in breast cancer patients and (B) lung cancer patients, subdivided by cancer study.

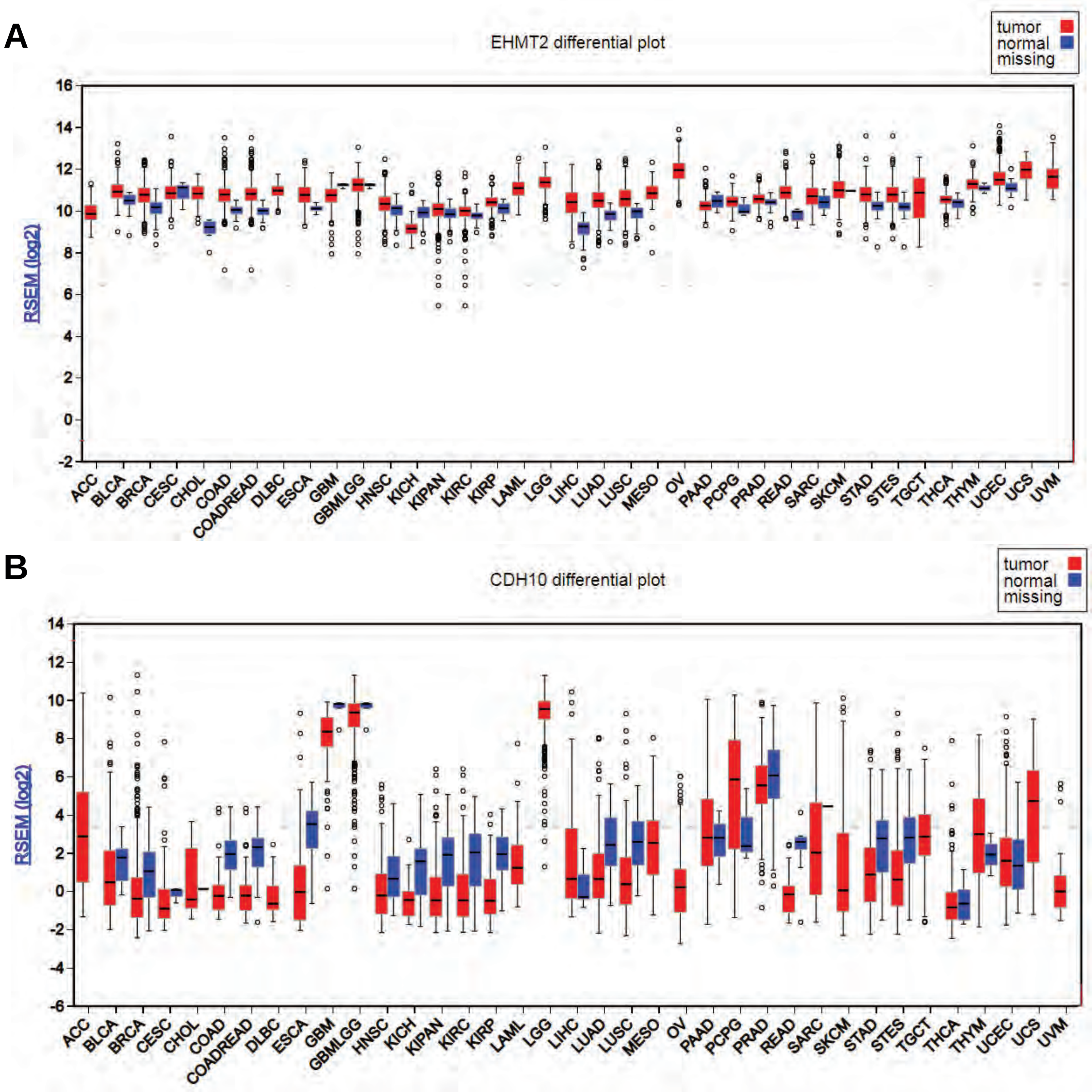

Figure S8. (A) TCGA Pan-cancer data comparing the expression levels of *EHMT2* in tumor and normal tissue samples. (B) TCGA Pan-cancer data comparing the expression levels of *CDH10* in tumor and normal tissue samples.

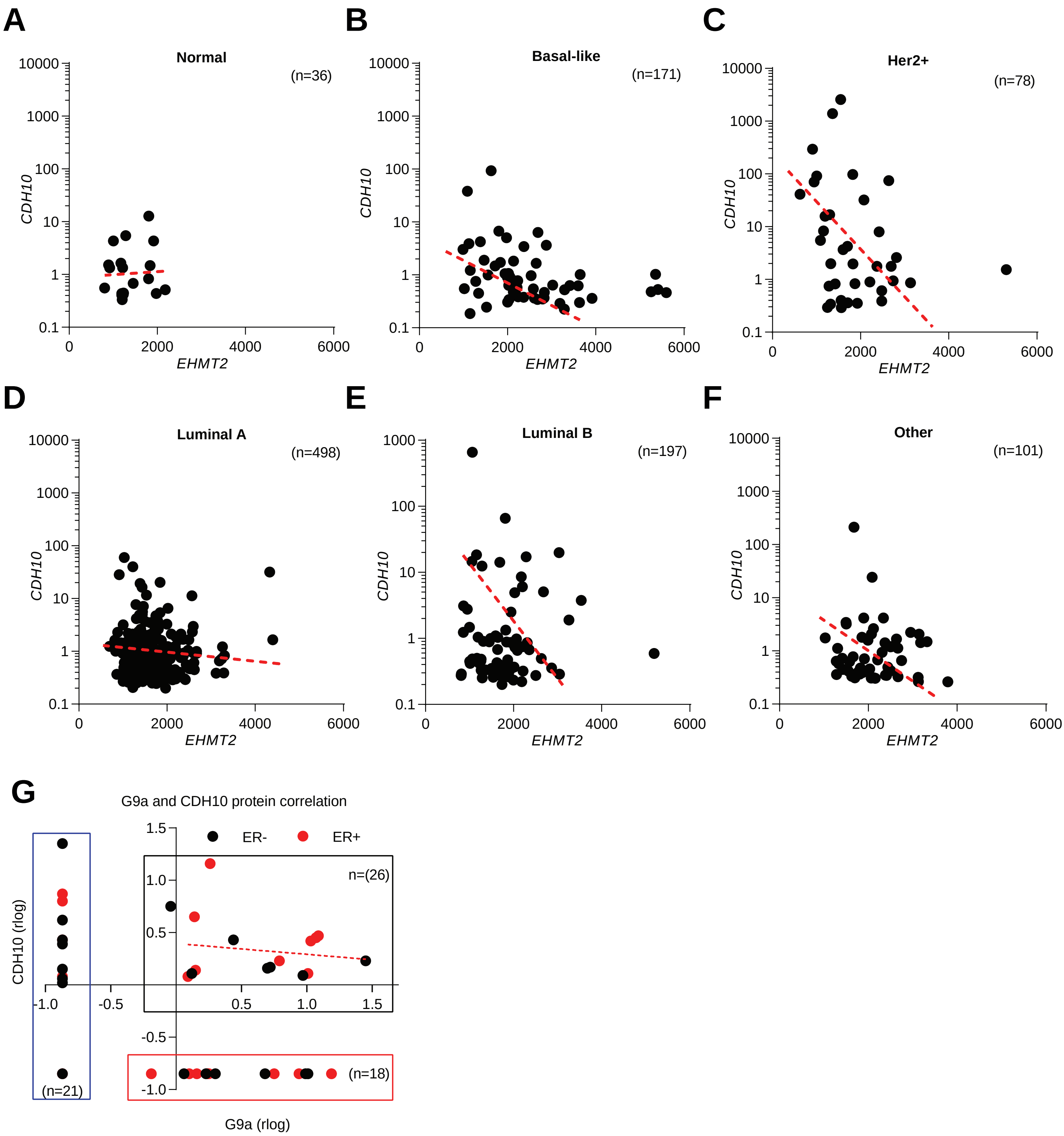

Figure S9. Correlation between *CDH10* and *EHMT2* (G9a) mRNA levels in (A) normal, (B) basal-like, (C) Her2+, (D) Luminal A, (E) Luminal B and (F) others in patient samples. (G) Correlation between G9a and CDH10 protein levels in breast cancer patients using publicly available databases. Blue square identifies patients with no detectable G9a. In the red square are patients with no detectable CDH10. In the black square are patients for which both proteins were detectable.

Table S1. Table S1: RT-PCR and ChIP primers

RT-PCR primers		
SIGLEC14	FWD	AGGATTTATTCTCCCATCTCGCT
	REV	GATGCTGATGGCGAGGTTCTG
IGSF5	FWD	CGGCGATGGCATCCTTCCTT
	REV	GACTCCGACATCTCCTCTTCAGGTAA
CDH10	FWD	GCAGTCCTGTTCCTGAGATTG
	REV	GCTGGCTTCTGCGAGCACACAGCG
CDH11	FWD	CCCGCCCCCCCGCCCCGCAC
	REV	CCCGGCCCCAGTCCCGGTCC
HPRT	FWD	TGACACTGGCAAAACAATGCA
	REV	GGTCCTTTTCACCAGCAAGCT
VEGF	FWD	CAGGTCAGAAACCAGCCAG
	REV	CGTGATGATTCAAACCTACC
IGFBP3	FWD	AGAGCACAGATACCCAGAACT
	REV	TGAGGAACTTCAGGTGATTCAGT
CEACAM7	FWD	TCAGCCTGTCCATACAGAGTG
	REV	TTGAACGGCACGACATCAATA
**ChIP primers**		
CDH10	FWD	GCAGTCCTGTTCCTGAGATTG
	REV	GCTGGCTTCTGCGAGCACACAGCG

Table S2. Pan-cancer analysis of CDH10 and EHMT2.

		N of samples			
		CDH10		EHMT2	
Abbreviation	Tumor type	Normal	Tumor	Normal	Tumor
READ	Rectum adenocarcinoma	10	40	10	167
COADREAD	Colorectal adenocarcinoma	50	134	51	626
COAD	Colon adenocarcinoma	40	94	41	459
LUAD	Lung adenocarcinoma	52	354	59	517
LUSC	Lung squamous cell carcinoma	49	321	51	501
BRCA	Breast invasive carcinoma	89	469	112	1100
STES	Stomach and Esophageal carcinoma	39	329	46	600
UCEC	Uterine Corpus Endometrial Carcinoma	29	359	35	546
KIRP	Kidney renal papillary cell carcinoma	28	79	32	291
KIRC	Kidney renal clear cell carcinoma	59	252	72	534
HNSC	Head and Neck squamous cell carcinoma	32	180	44	522
PRAD	Prostate adenocarcinoma	51	495	52	498
THYM	Thymoma	2	100	2	120
GBMLGG	Glioma	5	695	5	696
CESC	Cervical squamous cell carcinoma and endocervical adenocarcinoma	3	67	3	306
GBM	Glioblastoma multiforme	5	166	5	166
KICH	Kidney Chromophobe	20	24	25	66
